# Cross-cultural adaptation of the Pain Medication Questionnaire for use in Brazil

**DOI:** 10.1186/s12874-019-0821-x

**Published:** 2019-09-23

**Authors:** Sheila Raposo Galindo, Manoel Henrique da Nóbrega Marinho, Robert J. Gatchel, Tatiana de Paula Santana da Silva, Eduardo Henrique Soares Viana, Selene Cordeiro Vasconcelos, Murilo Duarte da Costa Lima

**Affiliations:** 1 Neuropsychiatry and Behavioral Sciences, Federal University of Pernambuco (UFPE), Av. João Cardoso Ayres, 480, Boa Viagem, Recife, Pernambuco Brazil; 2 State University of Pernambuco, UPE, Recife, Pernambuco Brazil; 3 University of Texas at Arlington, Arlington, USA; 4 Doctor Neuropsychiatry and Behavioral Sciences, Federal University of Pernambuco (UFPE), Recife, Pernambuco Brazil; 5 Systems Engineering, UPE, Recife, Pernambuco Brazil; 6 Neuroscience, Neuropsychiatry and Behavioral Sciences, Federal University of Paraíba (UFPB), João Pessoa, Paraíba Brazil; 7 Psychiatry, Neuropsychiatry and Behavioral Sciences Graduate Program, UFPE, Recife, Pernambuco Brazil

**Keywords:** Nursing, Opioids, Sickle cell anemia, Chronic pain, Surveys and questionnaires, Validation study, Mental health

## Abstract

**Background:**

The Pain Medication Questionnaire (PMQ) assesses the risk of opioid abuse in people with non-oncological chronic pain.

**Methods:**

This is a methodological study conducted at a hemotherapy centre in Recife, Pernambuco state, Brazil. A Cross-cultural adaptation was carried out by a committee of nine specialists, and we applied the PMQ to a pre-final sample of 40 individuals with sickle cell anemia, in addition to a sociodemographic and clinical questionnaire.

**Results:**

The mean agreement indexes for PMQ equivalences were the following: semantic (0.996), idiomatic (0.970), experiential (0.991), conceptual (0.953), language clarity (0.991), practical relevance (0.906), and theoretical relevance (0.945). Assessment of the PMQ showed that 50% of participants obtained a score equivalent to medium risk of opioid abuse. Cronbach’s alpha coefficient for the adapted PMQ instrument was 0.705, ranging from 0.641 to 0.736 among its items.

**Conclusion:**

The cross-cultural adaptation of the Pain Medication Questionnaire was satisfactory and easy to apply in the Brazilian population. It is clinically relevant, contributing professional practice and enlightening patients with sickle cell anemia on their behavioral dynamics with respect to opioid consumption. It will also contribute to teaching and research, because it is a useful tool for investigating the risk of abusive behavior in people with chronic pain.

**Electronic supplementary material:**

The online version of this article (10.1186/s12874-019-0821-x) contains supplementary material, which is available to authorized users.

## Background

Sickle cell anemia, a hereditary hemoglobinopathy caused by an important specific molecular lesion, is the conversion of glutamic acid to valine in the hemoglobin beta chain’ sixth position, giving rise to hemoglobin S [1]. When we deoxygenated hemoglobin it forms polymers that alter the cytoplasmic content of red blood cells, on which has a deformation phenomena, exhibiting an elongated sickle shape characterized by predominance of hemoglobin S [2, 3].

Recurring bouts of pain are the main complaint of patients with sickle cell anemia,

who frequently require emergency services, sometimes leading to hospitalization and death [4] and are more susceptible to significant morbidity and mortality [5, 6]. All the symptoms of sickle cell anemia are consequences of two primary physiopathological events. The first is vaso-occlusion with ischemia-reperfusion injury, which involves the narrowing of microcirculation caused by compacted red and white blood cells.

This creates vascular obstruction and tissue ischemia, triggering a complex series of events leading to tissue injury and intracellular accumulation of calcium. The second, hemolytic anemia, is the rupturing of red blood cells [7]. The pain’s symptoms that these patients experience in their lifetimes and their physiopathological mechanisms have yet to be well characterized [8, 9]. Vaso-occlusive crises belong to the group of nociceptive pain; however, the pain may be non-vaso-occlusive, and must therefore be investigated [10]. To that end, the consumption of opioids, sum up with the risk of abusive use of this analgesic by sickle cell anemia patients, makes it essential to assess behavior related to their opioid therapy by applying assessment instruments designed specifically for this purpose.

Research shows the increasing demand for translated instruments involving other cultural contexts, in order to perform intercultural comparison studies between different populations [11]. Cross-cultural adaptation involves a series of rigorous methodological steps, in order to ensure that the measuring aspects of the instrument are reliable and that they do not become distorted from the reality to which it will be adapted [12].

Furthermore, there are no limitations about the usage of cross-cultural instruments’ adaptations over time. As such, new cross-cultural adaptations are possible and generally necessary for better adaptation to the target population [13].

The need to select a rigorous protocol to assess the conceptual equivalence of semantic, operational, measuring and functional aspects between the original instrument and the adapted version is justified by the need for conceptual clarity and the large number of ways to perform the different types of equivalence. There is also a clear need to standardize definitions and operationalize these equivalences [13].

The original Pain Medication Questionnaire (PMQ) was developed for this study and validated in the USA by (Additional file 1) to assess the risk of opioid abuse in patients with chronic non-oncological pain in a series of four studies [14–17]. However, it was only adapted for Italian [18], and is being submitted to the entire cross-cultural adaptation process in this study, as well as clinical validation in a subsequent investigation, both carried out by the research team. As such, the aims of the present study were to transculturally-adapt and check the content and face validity of the PMQ for use in Brazil.

We believe that the PMQ will help health professionals in planning more effective interventions to manage opioid consumption by individuals with heterogeneous chronic pain, contributing to understanding the behavioral dynamics of these users. Furthermore, it will aid in the teaching and research of this phenomenon, given that it is an important measuring instrument capable of guiding treatment decisions.

## Method

### Subject recruitment and assessment

The study uses consecutive intentional non-probability sampling, adopting the following inclusion criteria: opioid-using patients of both sexes with sickle cell anemia, aged between 18 and 59 years, undergoing treatment at a hemotherapy service in Pernambuco state, Brazil. Excluded were individuals with any clinical and/or psychic conditions that hindered instrument application.

The simplest way to recalculate cutoffs based on the normative scores in this dataset is to use the mean (average) score and the standard deviation (measure of spread). It is suggested that a five-point scale of overall risk be used in place of a three-point scale, as this provides a greater range and therefore more accuracy. By this method, respondents are classified as very low, low, average, high, or very high risk. However, if desired, a three-level classification could be constructed by taking a midpoint between the very low/low and high/very high categories.

The following calculations were used to identify the cutoffs points: very low: mean score – 1.5 x standard deviation; low: mean score - 0.75 x standard deviation; high: mean score + 0.75 x standard deviation; very high: mean score + 1.5 x standard deviation, as per original article.

The cut-off points were classified as: very low (average score of – 1.5 x standard deviation); low (average score of – 0.75 x standard deviation); high (average score of + 0.75 x standard deviation); and very high, (average score of + 1.5 x standard deviation). Thus, the subjects were classified as having cutoff points for each risk level of the PMQ, ranging from very low (<18); low (18–34); medium (35–42); high (43–50) and very high (>50).

### Cross-cultural adaptation of the original version (Additional file 1) of the pain medication questionnaire

We use methodological study on the cross-cultural adaptation of the PMQ for use in Brazil, conducted according to the [13, 19] protocol, described below:

- Step 1 – Initial translation

This step consists of two independent translations from English to Portuguese. One translator (T1) was not from the health area and was unaware of the aim of the translation; the second translator (T2), a health professional, was informed of the objective of the translation. The translators were native Portuguese speakers fluent in English. The two translators (T1 and T2) presented a report detailing the difficulties and doubts encountered with the PMQ items.

- Step 2 – Synthesis of the translations

This step synthetizes the results of the two translations, with the aid of the original PMQ (English), the translations and reports from Step 1. This synthesis was performed by the researcher, recorded and discussed with the research team. Adjustments were then made and the synthetized version of the instrument (T12) was constructed for use in next Step.

- Step 3 – Back translation to the original language

Back translation was performed by two double blind translators who were unaware of the study objectives. The translators, native English speakers fluent in the target language (Portuguese), carried out back translations “BT1 and BT2”, aimed at detecting possible flaws in the synthesis stage of PMQ (construction of T12) and checking content validity, since it identifies serious inconsistencies or conceptual errors in the initial translations [19].

- Step 4 – Revision by a committee of specialists

Two translations (T1 and T2), synthetized translations (T12) and the two back translations (BT1 and BT2), in addition to the original version of the PMQ instrument, were sent to a committee of nine specialists. The criteria established by [20] with some adaptations.

- Step 5 – Revision by a committee of specialists

Were used to select these PMQ validated by the specialists, is to check the comprehension of sickle anemia patients with chronic pain regarding opioid use, their difficulties in understanding the instruments as well as their suggestions, in order to.

validate it for the target population (face and content validity). This step is vital to semantic analysis and face validity, since it most approximates the day-to-day language used by the target population and investigates errors in understanding the items [21, 22].

The PMQ is self-applied, but it is mandatory for its participants were asked to read the instrument in order to reduce the time spent on filling it out and resolve possible reading problems due to the low schooling levels of the subjects. The sample size at this stage (pre-final sample) was based on [19], who recommend that the translated version be applied to a sample of 30 to 40 individuals from the target population. In this study, 40 participants completed the PMQ at the hemotherapy center of Pernambuco, reporting their perceptions of the meaning of each item and the scoring method. We recorded the time participants required to complete the instrument, as well as their difficulties, observations and suggestions.

### Analysis

#### Descriptive statistics

We analyse the data descriptively using measures of central tendency and dispersion, and analyzed by applying the arithmetic mean, standard deviation and median, in addition to absolute frequencies and percentages. Fisher’s exact test, a statistical significance test used to analyze the independent observations of two or more random variables, was applied to assess the occurrence of a significant association between PMQ classification and the categorical variables related to sociodemographic profile.

The significance level of the statistical tests was set at 5% (*p*-value = 0.05). IBM-SPSS (Statistical Package for Social Science) software, version 23 [23] was used to digitize the data and perform statistical calculations.

For the specialist assessment step, the relative frequency of satisfactory responses was obtained in each of the items evaluated. The satisfactory answers in the items related to language clarity, and theoretical and practical relevance were “very” and “extremely”. In equivalence assessment (semantic, idiomatic, experiential and conceptual) the satisfactory answer was “I agree”. In assessment of relevance the satisfactory answers were “Yes”, “Really relevant” or “Very relevant”.

To determine the reliability of the adapted PMQ for use in Brazil, internal consistency was obtained for the pre-final sample using the Cronbach alpha technique [24], and the influence of each item according to Spearman’s coefficient correlation was analyzed.

#### Face validity of the PMQ assessed by the specialists and the target population in the pre-final sample

To determine face validity, participants answer the following question to determine face validity: “Does the adapted PMQ instrument assess the risk of opioid abuse in people with chronic pain?” All subjects answered yes. It is important to underscore that the concept of heterogeneous chronic pain was explained to the target population.

#### Content validity of the PMQ assessed by the specialists and the target population in the pre- final sample

The average agreement index (AI) of all the items (Tables 1 and 2) was calculated by the specialists and target population for language clarity (0.991), practical relevance (0.906) and theoretical relevance (0.945), obtaining satisfactory results. However, since items 4 (0.333), 11(0.667) and 26 (0.667) received below average scores for practical relevance and items 11 (0.667) and 26 (0.667) for theoretical relevance, it was suggested that changes be made to these items.

**Table 1:** Assessment of content validation of the adapted PMQ for language clarity, practical relevance and theoretical relevance

PMQ items	Language clarity	Practical relevance	Theoretical relevance
1	1.000	1.000	1.000
2	1.000	0.778	1.000
3	1.000	1.000	1.000
4	0.889	0.333	0.889
5	1.000	1.000	1.000
6	1.000	1.000	1.000
7	1.000	1.000	1.000
8	1.000	1.000	1.000
9	1.000	1.000	1.000
10	1.000	0.778	0.778
11	1.000	0.667	0.667
12	1.000	1.000	1.000
13	1.000	1.000	1.000
14	1.000	0.778	1.000
15	1.000	1.000	1.000
16	1.000	1.000	1.000
17	1.000	1.000	1.000
18	1.000	1.000	1.000
19	1.000	0.778	0.778
20	1.000	0.778	0.778
21	1.000	1.000	1.000
22	1.000	1.000	1.000
23	1.000	1.000	1.000
24	1.000	1.000	1.000
25	1.000	1.000	1.000
26	0.889	0.667	0.667
Average	0.991	0.906	0.945

Source: the authors

**Table 2:** Assessment of equivalences in the pre-final version of the PMQ

PMQ items	Semantic equivalence	Idiomatic equivalence	Experiential equivalence	Conceptual equivalence
1	1.000	1.000	1.000	1.000
2	1.000	1.000	1.000	1.000
3	1.000	1.000	1.000	1.000
4	1.000	1.000	1.000	1.000
5	1.000	1.000	1.000	1.000
6	1.000	1.000	1.000	1.000
7	1.000	1.000	1.000	0.889
8	1.000	1.000	1.000	1.000
9	1.000	1.000	1.000	1.000
10	1.000	1.000	1.000	0.667
11	1.000	1.000	1.000	1.000
12	1.000	1.000	0.889	0.778
13	1.000	1.000	1.000	1.000
14	1.000	1.000	1.000	0.889
15	1.000	1.000	1.000	0.889
16	1.000	1.000	1.000	1.000
17	0.889	0.889	0.889	0.889
18	1.000	1.000	1.000	1.000
19	1.000	1.000	1.000	1.000
20	1.000	1.000	1.000	1.000
21	1.000	1.000	1.000	1.000
22	1.000	1.000	1.000	1.000
23	1.000	1.000	1.000	0.889
24	1.000	0.778	1.000	0.889
25	1.000	0.778	1.000	1.000
26	1.000	0.778	1.000	1.000
Average	0.996	0.970	0.991	0.953

Source: the authors

The specialists’ evaluation of semantic (0.996), idiomatic (0.970), experiential (0.991) and conceptual equivalence (0.953) showed satisfactory results; however, changes to item 10 (0.667) were suggested, since it scored below average.

Thus, items 7, 10, 11, 12, 17, 23, 24, 25 and 26 were changed in accordance with the specialists’ suggestions. Item 4 was not changed and the final pre-final version of the PMQ is shown in Table 3.

**Table 3:** Comparison between the original PMQ version(Adams, 2004) and the adapted translated and back translation versions

Item	PMQ – original scale	PMQ- adapted translated version (pre-final)	Back translation
i1	I believe I am receiving enough medication to relieve my pain.	Eu acredito estar recebendo medicação suficiente para aliviar minha dor.	I believe I am receiving enough medication to relieve my pain.
i2	My doctor spends enough time talking to me about my pain medication during appointments.	Meu médico passa tempo suficiente falando comigo sobre minha medicação para dor durante as consultas.	My doctor spends enough time talking to me about my pain medication during appointments.
i3	I believe I would feel better with a higher dosage of my pain medication.	Eu acredito que me sentiria melhor com uma dosagem maior da minha medicação para dor.	I believe I would feel better with a higher dosage of my pain medication.
i4	In the past, I have had some difficulty getting the medication I need from my doctor(s).	No passado, eu tive algumas dificuldades em conseguir a medicação que eu precisava do(s) meus(s) médicos(s).	In the past, I have had some difficulty getting the medication I need from my doctor(s).
i5	I wouldn’t mind quitting my current pain medication and trying a new one, if my doctor recommends it.	Eu não me importaria em parar minha atual medicação para dor e tentar uma nova, se meu médico me recomendasse isso.	I wouldn’t mind quitting my current pain medication and trying a new one, if my doctor recommends it.
i6	I have clear preferences about the type of pain medication I need.	Eu tenho claras preferências sobre o tipo de medicação que preciso para dor.	I have clear preferences about the type of pain medication I need.
i7*	Family members seem to think that I may be too dependent on my pain medication.	Pessoas da família parecem achar que eu posso estar muito dependente da minha medicação para dor.	People members seem to think that I may be too dependent on my pain medication.
i8	It is important to me to try ways of managing my pain in addition to the medication (such as relaxation, biofeedback, physical therapy, TENS unit, etc.).	É importante para mim, testar formas adicionais à minha medicação para administrar minha dor como: relaxamento, biofeedback, fisioterapia, uso de TENS (Estimulação Elétrica Nervosa Transcutânea), etc.	It is important to me to try ways of managing my pain in addition to the medication (such as relaxation, biofeedback, physical therapy, TENS unit, etc.).
i9	At times, I take pain medication when I feel anxious and sad, or when I need help sleeping.	Às vezes, eu tomo medicação para dor quando eu me sinto ansioso(a) e triste, ou quando preciso de ajuda para dormir.	At times, I take pain medication when I feel anxious and sad, or when I need help sleeping.
i10*	At times, I drink alcohol to help control my pain.	Às vezes, eu tomo bebida alcoólica para ajudar a controlar minha dor.	At times, I drink alcohol to help control my pain.
i11*	My pain medication makes it hard for me to think clearly sometimes.	Algumas vezes, minha medicação para dortorna difícil para mim pensar com clareza.	My pain medication makes it hard for me to think clearly sometimes.
i12*	I find it necessary to go to the emergency room to get treatment for my pain.	Eu acho necessário ir a um serviço de urgência para conseguir tratamento para minha dor.	I find it necessary to go to the emergency service to get treatment for my pain.
i13	My pain medication makes me nauseated and constipated sometimes.	Minha medicação para dor às vezes me deixa enjoado e constipado.	My pain medication makes me nauseated and constipated sometimes.
i14	At times, I need to borrow pain medication from friends or family to get relief.	Às vezes, eu preciso pedir emprestado aos meus amigos ou familiares medicação para ter alívio.	At times, I need to borrow pain medication from friends or family to get relief.
i15	I get pain medication from more than one doctor in order to have enough medication for my pain.	Eu pego medicação para dor em mais de um médico a fim de ter medicação suficiente para minha dor.	I get pain medication from more than one doctor in order to have enough medication for my pain.
i16	At times, I think I may be too dependent on my pain medication.	Às vezes, eu acho que posso está muito dependente da minha medicação para dor.	At times, I think I may be too dependent on my pain medication.
i17*	To help me out, family members have obtained pain medications for me from their own doctors.	Para me ajudar, pessoas da família obtêm medicamento para dor para mim, de seus próprios médicos.	To help me out, family people have obtained pain medications for me from their own doctors.
i18	At times, I need to take pain medication more often than it is prescribed in order to relieve my pain.	Às vezes, eu tenho que tomar medicação para dor com mais frequência do que está prescrito, a fim de aliviar minha dor.	At times, I need to take pain medication more often than it is prescribed in order to relieve my pain.
i19	I save any unused pain medication I have in case I need it later.	Eu guardo qualquer medicação para dor que não usei, caso precise dela mais tarde.	I save any unused pain medication I have in case I need it later.
i20	I find it helpful to call my doctor or clinic to talk about how my pain medication is working.	Eu acho útil ligar para meu médico ou para a clínica para falar sobre como meu medicamento para dor está agindo.	I find it helpful to call my doctor or clinic to talk about how my pain medication is working.
i21	At times, I run out of pain medication early and have to call my doctor for refills.	Às vezes, meus medicamentos acabam antecipadamente e eu tenho que ligar para meu médico para reabastecer.	At times, I run out of pain medication early and have to call my doctor for refills.
i22	I find it useful to take additional medications (such as sedatives) to help my pain medication work better.	Eu acho útil tomar medicamentos adicionais (como sedativos) para ajudar minha medicação para dor funcionar melhor.	I find it useful to take additional medications (such as sedatives) to help my pain medication work better.
i23*	How many painful conditions (injured body parts or illnesses) do you have?	Quantas partes do corpo doloridas (partes do corpo lesionadas ou enfermidade) você tem?	How many painful conditions (injured body parts or illnesses) do you have?
i24*	How many times in the past year have you asked your doctor to increase your prescribed dosage of pain medication in order to get relief?	Quantas vezes, no último ano, você pediu para o seu médico para aumentar a dosagem prescrita de medicação para dor a fim de ter alívio?	How many times in the last year have you asked your doctor to increase your prescribed dosage of pain medication in order to get relief?
i25*	How many times in the past year have you run out of pain medication early and had to request an early refill?	Quantas vezes, no último ano, você ficou sem medicação para dor antecipadamente e teve que pedir um reabastecimento antecipado?	How many times in the last year have you run out of pain medication early and had to request an early refill?
i26*	How many times in the past year have you accidentally misplaced your prescription for pain medication and had to ask for another?	Quantas vezes, no último ano, você acidentalmente perdeu sua prescrição de medicação para dor e teve que pedir outra?	How many times in the last year have you accidentally misplaced your prescription for pain medication and had to ask for another?

Source: the authors

#### Semantic assessment by the target population

All participants in the pre-final sample agreed with the semantics of the adapted PMQ version for use in Brazil, all exhibited good comprehension, suggested no changes and expressed no difficulty in understanding the items.

#### Original PMQ, pre-final adapted translated version and back translation

The adapted translated version of the PMQ showed a satisfactory translation and back translation due to the adjustments made to the cross-cultural adaptation for Brazil, based on suggestions from the committee of specialists and target population. After the translation (T12), a number of linguistic changes were made to more accurately reflect the Brazilian culture and achieve better cross-cultural adaptation.

#### PMQ reliability based on data collected in the pre-final sample

Indications about PMQ’s factorial analysis in its original version show eight potential factors (forming eight sub-factors), most of which correlated positively and, based on the results of factorial analysis, can be combined without losing accuracy or reliability. However, one factor was negatively correlated with the other factors.

In the score calculation guide, the author suggests removing this factor in order to increase reliability. This factor contains the following items:

- Item 5: I wouldn’t mind quitting my current pain medication and trying a new one, if my doctor recommends it.
- Item 6: I have clear preferences about the type of pain medication I need.
- Item 8: It is important to me to try ways of managing my pain in addition to the medication (such as relaxation, biofeedback, physical therapy, TENS (transcutaneous nervous electrical stimulation) etc. [15].

Internal consistency and reliability of the pre-final PMQ instrument were checked by Cronbach’s alpha, and values higher than 0.700 are acceptable [25, 26]. The intensity of the correlation between the items of a measuring instrument can be determined if this coefficient increases after eliminating an item from the instrument. If this occurs, it can be assumed that the item is not highly correlated with the others on the scale.

On the other hand, if the coefficient decreases it can be inferred that the item is highly correlated with the other items on the scale. Thus, Cronbach’s alpha determines whether the scale is actually reliable, since it assesses how each item reflects its reliability.

Cronbach’s alpha was initially assessed for the 26 items, obtaining a coefficient of The total scores ranged from 26 to 70, with an average of 43.35, standard deviation of 10.46 and median of 40.50. According to the author’s suggestion and technical analysis, items 5, 6 and 8 were excluded from the instrument and the scores of items 1 and 2 were inverted. The Cronbach’s alpha value obtained for the other 23 items was 0.705, considered acceptable in the initial study phase of a new instrument, with gains of around 15%. The total scores of the 23 items varied between 17 and 62, with an average of 34.63, standard deviation of 10.71 and median of 34.

The alpha values were assessed by excluding each item in order to identify possible flaws in the internal consistency of the instrument. Gains were observed for each item, as shown in Table 4, with the minimum obtained for item 2 (11.50%) and maximum for item 1 (19.50%). The average increase for the 23 items was 15.10%, demonstrating that the exclusion of items 5, 6 and 8 produced satisfactory results. Moreover, Spearman’s correlation coefficient was calculated to determine the influence of each item on the internal consistency of the PMQ items, varying from − 0.132 to 0.802 (Table 4).

**Table 4:** Spearman’s correlation and Cronbach’s alpha of each item with the PMQ score (between the 23 and 26 items)

Items	Correlation (23)	Alpha if the item was not detected (26)	Alpha if the item was detected (23)	Increase (%)
P1 - I believe I am receiving enough medication to relieve my pain.^b^	0.802^a^	0.516	0.641	19.50%
P2 - My doctor spends enough time talking to me about my pain medication during appointments. ^b^	−0.072	0.651	0.736	11.55%
P3 - I believe I would feel better with a higher dosage of my pain medication.	0.653^a^	0.576	0.687	16.16%
P4 - In the past, I have had some difficulty getting the medication I need from my doctor(s).	0.348^a^	0.615	0.717	14.23%
P7 –Family members seem to think that I may be too dependent on my pain medication.	0.485^a^	0.581	0.686	15.31%
P9 - At times, I take pain medication when I feel anxious and sad, or when I need help sleeping.	0.370^a^	0.585	0.69	15.22%
P10 – At times, I drink alcohol to help control my pain.	0.284	0.605	0.703	13.94%
P11 – My pain medication makes it hard for me to think clearly sometimes.	0.308	0.597	0.693	13.85%
P12 – I find it necessary to go to the emergency room to get treatment for my pain.	0.586^a^	0.582	0.685	15.04%
P13 – My pain medication makes me nauseated and constipated sometimes.	0.1	0.627	0.719	12.80%
P14 – At times, I need to borrow pain medication from friends or family to get relief.	0.387^a^	0.582	0.686	15.16%
P15 – I get pain medication from more than one doctor in order to have enough medication for my pain.	0.534^a^	0.581	0.687	15.43%
P16 – At times, I think I may be too dependent on my pain medication.	0.434^a^	0.578	0.687	15.87%
P17 – To help me out, family members have obtained pain medications for me from their own doctors.	0.443^a^	0.581	0.687	15.43%
P18 – At times, I need to take pain medication more often than it is prescribed in order to relieve my pain.	0.492^a^	0.557	0.672	17.11%
P19 – I save my unused pain medication I have in case I need it later.	−0.132	0.629	0.723	13.00%
P20 – I find it helpful to call my doctor or clinic to talk about how my pain medication is working.	0.177	0.598	0.702	14.81%
P21 – At times, I run out of pain medication early and have to call my doctor for refills.	0.566^a^	0.568	0.679	16.35%
P22 – I find it useful to take additional medications (such as sedatives) to help my pain medication work better.	0.142	0.602	0.713	15.57%
P23 – How many painful conditions (injured body parts or illnesses) do you have?	0.075	0.623	0.721	13.59%
P24 – How many times in the past year have you asked your doctor to increase your prescribed dosage of pain medication in order to get relief?	0.326^a^	0.594	0.699	15.02%
P25 – How many times in the past year have you run out of pain medication early and had to request an early refill?	0.623^a^	0.549	0.666	17.57%
P26 – How many times in the past year have you accidentally misplaced your prescription for pain medication and had to ask for another?	0.418^a^	0.59	0.692	14.74%

Source: the authors

^a^Statistically different from zero

^b^The scale scores were inverted

## Results

### PMQ items

Table 5 contains 26 PMQ items, underscoring the percentages of a number of the answers. Only 17.50% totally disagreed with item i2 (“My doctor spends enough time talking to me about my pain medication during appointments”.)

**Table 5:** Results of PMQ items, according to the pre-final sample. Recife-Pernambuco, Brazil, 2015–2017

Item	Answer									
	0		1		2		3		4	
	n	% ^a^	n	%	n	%	N	%	n	%
P1	17	42.50	–	–	–	–	3	7.50	20	50.00
P2	7	17.50	2	5.00	–	–	14	35.00	17	42.50
P3	5	12.50	1	2.50	–	–	11	27.50	23	57.50
P4	7	17.50	3	7.50	–	–	12	30.00	18	45.00
P5	12	30.00	1	2.50	2	5.00	5	12.50	20	50.00
P6	11	27.50	–	–	–	–	4	10.00	25	62.50
P7	27	67.50	–	–	2	5.00	4	10.00	7	17.50
P8	5	12.50	–	–	–	–	7	17.50	28	70.00
P9	36	90.00	1	2.50	2	5.00	–	–	1	2.50
P10	37	92.50	3	7.50	–	–	–	–	–	–
P11	17	42.50	6	15.00	7	17.50	1	2.50	9	22.50
P12	–	–	3	7.50	18	45.00	13	32.50	6	15.00
P13	4	10.00	7	17.50	18	45.00	4	10.00	7	17.50
P14	19	47.50	4	10.00	10	25.00	6	15.00	1	2.50
P15	10	25.00	8	20.00	16	40.00	3	7.50	3	7.50
P16	27	67.50	2	5.00	–	–	5	12.50	6	15.00
P17	31	77.50	4	10.00	–	–	3	7.50	2	5.00
P18	15	37.50	9	22.50	10	25.00	2	5.00	4	10.00
P19	1	2.50	–	–	2	5.00	2	5.00	35	87.50
P20	28	70.00	8	20.00	1	2.50	–	–	3	7.50
P21	21	52.50	8	20.00	4	10.00	2	5.00	5	12.50
P22	12	30.00	–	–	19	47.50	3	7.50	6	15.00
P23	6	15.00	7	17.50	13	32.50	6	15.00	8	20.00
P24	24	60.00	7	17.50	5	12.50	1	2.50	3	7.50
P25	14	35.00	6	15.00	8	20.00	9	22.50	3	7.50
P26	27	67.50	10	25.00	2	5.00	–	–	1	2.50

Source: the authors

^a^ The percentages were obtained based on the answers of the 40 participants

In item i6, 62.50% agreed with “I have clear preferences about the type of pain medication I need”, revealing a strong inclination for certain medications, as well as in i3, where 57.50% agreed with “I believe I would feel better with a higher dosage of my pain medication”, expressing dissatisfaction with the prescribed dosage, and reinforcing the answers of item i1 (“I believe I am receiving enough medication to relieve my pain”), where 42.50% of the target population totally disagreed. All the participants go to the emergency service to treat their pain.

A little over half (57.50%) agreed, at varying intensities, with i11 (“My pain medication makes it hard for me to think clearly sometimes”). Nearly all the sample (90%) agreed, at different intensities, with i13 (“My pain medication makes me nauseated and constipated sometimes”).

However, in i24 (“How many times in the last year have you asked your doctor to increase your prescribed dosage of pain medication in order to get relief?”), 60% of the target population denied having done so.

Most (67.50%) disagreed with item i7 (“Family members seem to think that I may be too dependent on my pain medication”), implying a low score. Similarly, most of the subjects (67.50%) denied i16 (“At times, I think I may be too dependent on my pain medication”). In item i8 (“It is important to me to try ways of managing my pain in addition to the medication, such as relaxation, biofeedback, physical therapy, use of TENS (transcutaneous electrical nervous stimulation), etc”), there was 70% agreement in accepting other forms of pain management, such as adjuvant therapy. In i5, the majority agreed, at different intensities, with the statement “I wouldn’t mind quitting my current pain medication and trying a new one, if my doctor recommends it”, and only 30% totally disagreed.

Most (90%) denied item i9 (“At times, I take pain medication when I feel anxious and sad, or when I need help sleeping”), i10 (“At times, I drink alcohol to help control my pain”) (92.50%), and i17 (To help me out, family members have obtained pain medications for me from their own doctors“) (77.50%).

In item i19 (“I save my unused pain medication I have in case I need it later”) 87.50% answered always, in order to store medications, which is reinforced by item i4, where many agreed, at varying intensities, that in the past they experienced difficulty obtaining the medication they needed from their doctor and a minority disagreed (17.50%) with this fact.

Also reinforcing these statements, 75% confirmed, at different intensities, item i15 (“I get medication from more than one doctor in order to have enough medication for my pain”.) Item i20 (“I find it helpful to call my doctor or clinic to talk about how my pain medication is working”) was denied by 70%. Item i21 (“At times, I run out of pain medication early and have to call my doctor for refills”) was denied by 52.50%, although 20% called infrequently, 10% at times, 5% frequently and 12.50% always called asking for more medication, not demonstrating excessive focus when speaking about their pain medication. A little over half (52.50%) confirmed, at varying intensities, item i14 (“At times, I need to borrow pain medication from friends or family to get relief”.)

Also related to obtaining pain medication, 62.50% confirmed i18 (“At times, I need to take pain medication more often than it is prescribed in order to relieve my pain”) at different intensities. By contrast, in i25 (“How many times in the past year have you run out of pain medication early and had to request an early refill?”), only 35% denied and the rest confirmed running out of medication early and asking for refills at varying frequencies. In item i26 (“How many times in the past year have you accidentally misplaced your prescription for pain medication and had to ask for another?”), 67.50% denied doing so.

A large number of different agreement levels (70%) can be observed for item i22 (“I find it useful to take additional medications, such as sedatives, to help my pain medication work better”). In i23 (“How many painful conditions (injured body parts or illnesses) do you have?”), 32.50% answered having 3 painful body parts.

#### Sociodemographic and clinical aspects

In Table 6, the sociodemographic questionnaire shows that the pre-final sample was composed of 62.50% women and 37.50% men, most (67.50%) from the emergency sector and the outpatient facility (32.50%), with an age range between 18 and 53 years, average of 31.03 years, standard deviation of 8.89 years and median of 29 years, and a predominance of brown-skinned individuals (50%) and blacks (45%), 30% with incomplete elementary schooling and 35% unemployed.

**Table 6:** Sociodemographic characteristics of the pre-final sample correlated with the PMQ. Recife- Pernambuco, Brazil, 2015–2017

Variable	PMQ classification						*p*-value
	Low		Medium		High		
	N	%	n	%	N	%	
HEMOPE (Blood Center of Pernambuco)/emergency sector	5	18.50	16	59.30	6	22.20	*p*^b^ = 0,105
Outpatient	7	53.80	4	30.80	2	15.40	
Age range (years)							*p*^b^ = 0.346
18 to 29	4	19.00	12	57.10	5	23.80	
30 to 53	8	42.10	8	42.10	3	15.80	
Sex							*p*^b^ = 0.691
Male	4	26.70	9	60.00	2	13.30	
Female	8	32.00	11	44.00	6	24.00	
Color							*p*^b^ = 0.229
White	0	0.00	1	50.00	1	50.00	
Black	3	16.70	11	61.10	4	22.20	
Brown	9	45.00	8	40.00	3	15.00	
Marital status							*p*^b^ = 0.206
Married/common law	6	54.50	4	36.40	1	9.10	
Single	4	19.00	13	61.90	4	19.00	
Separated/divorced	2	25.00	3	37.50	3	37.50	
Schooling							*p*^b^ = 0.391
Incomplete elementary	6	50.00	4	33.30	2	16.70	
Elementary	2	15.40	7	53.80	4	30.80	
Secondary	3	25.00	8	66.70	1	8.30	
University	1	33.30	1	33.30	1	33.30	
Occupation							*p*^b^ = 0.025^a^
Student	–	–	1	33.30	2	66.70	
Casual labor	4	57.10	2	28.60	1	14.30	
Unemployed	3	21.40	8	57.10	3	21.40	
Formally employed	2	66.70	–	–	1	33.30	
Homemaker	2	100.00	–	–	–	–	
Others	1	9.10	9	81.80	1	9.10	
Current religion							*p*^b^ = 0.825
Catholic	2	20.0	6	60.00	2	20.00	
Evangelical	9	39.1	9	39.10	5	21.70	
None	1	20.0	3	60.00	1	20.00	
Others	0	0.0	2	100.00	0	0.00	

Source: the others

^a^ Significant association at 5%

^b^ Fisher Exact test

According to the PMQ, the percentage of low risk individuals was higher in the outpatient than the emergency group (53.80% × 18.50%), while the percentage at medium and high risk was larger in the emergency group (59.30% × 30.80% with an average risk and 22.20% × 15.40% with high risk). However, the association was not significant (*p*>0.05), because some emergency patients have a history of more frequent visits to the emergency service in search of pain medication compared to outpatients.

The 18–29 age range included 57.10% in the medium-risk group and 60% of the men and 44%% of the women were at medium risk, with blacks (61.10%) and those with secondary schooling (66.70%) predominating (Table 5). In the high-risk group 66.70% are represented by students, with a significant association (*p*<0.05).

Table 7 presents the sociodemographic variables related to the clinical picture of the patients with sickle cell anemia, classified according to the PMQ, demonstrating that most of the respondents from the pre-final sample (*n* = 33) reported feeling sad, depressed, dispirited or unable to experience pleasure, even from activities usually found enjoyable, with 48.50% obtaining significant scores on the PMQ. These individuals were classified as medium-risk, as were 43.30% of respondents (*n* = 30) who felt nervous, tense, unable to relax, worried or agitated.

**Table 7:** Assessment of PMQ classification according to the sociodemographic variables related to clinical picture. Recife- Pernambuco, Brazil, 2015–2017

Variable	PMQ classification						*p*-value
	Low		Medium		High		
	N	%	N	%	N	%	
P20^b^							*p*^a^ = 0.412
Yes	9	27.30	16	48.50	8	24.20	
No	3	42.90	4	57.10	–	–	
P21^c^							*p*^a^ = 0.150
Yes	9	30.00	13	43.30	8	26.70	
No	3	30.00	7	70.00	0	0.00	
P23^d^							*p*^a^ = 0.132
SBP	3	60.00	1	20.00	1	20.00	
Leg ulcers	0	0.00	6	85.70	1	14.30	
Unknown	9	32.10	13	46.40	6	21.40	
P24^e^							*p*^a^ = 0.366
Yes	4	25.00	7	43.80	5	31.30	
No	8	33.30	13	54.20	3	12.50	

Source: the authors

^a^Fisher Exact test

^b^“P20 – In the last two weeks (including today) have you felt troubled most of the day, because you were sad, depressed, dispirited or unable to experience pleasure, even from activities usually found enjoyable?”

^c^“P21 – In the last two weeks (including today) have you felt nervous, tense, unable to relax, worried or agitated?”

^d^“P23 – Besides sickle cell anemia, have you had any other disease?”

^e^“P24 – Does anyone else in the family have sickle cell anemia?”

### Score for risk of opioid abuse in people with heterogeneous chronic pain

According to the PMQ, 50% of the pre-final sample was classified as being at medium risk of opioid abuse and 20% at high/very high risk. There was a significant difference in the PMQ score between medium risk and very low/low and very high risk (Table 8).

**Table 8:** PMQ classification according to the pre-final sample. Recife- Pernambuco, Brazil, 2015–2017

PMQ classification (23 questions)	n	%
Very low / Low	12	30.00
Medium	20	50.00
High/Very High	8	20.00
TOTAL	40	100.00

Source: the authors

## Discussion

### Cross-cultural adaptation and face validity

Assessment of conceptual equivalence and the items showed that the PMQ is a trustworthy and practical instrument for application in sickle cell anemia patients suffering from chronic pain and using opioids in Brazil, which prompted the decision to proceed with its translation and cultural adaptation. Semantic assessment by the target population followed literature recommendations with respect to schooling levels, demonstrating that the items were understood by all members at whom the instrument is aimed [27, 28]. The cross-cultural adaptation of the PMQ exhibited face and content validity characteristics similar to those reported by [19].

### Sociodemographic characteristics

The sociodemographic characteristics of the pre-final sample in this study on the PMQ were similar to those of other investigations of patients with sickle cell anemia, highlighting the prevalence of single women, low schooling levels, poor physical condition and associated diseases. This corroborates the studies of [29] and [30], revealing a significant effect on a number of phases of life [3, 31–33]. There were no significant differences in age and sex for PMQ scores, similarly to that observed by [19].

The clinical questionnaire’s results showed behavioral changes related to mood disorders in a large number of patients with sickle cell anemia, demonstrating a correlation between opioid dependence and these disorders [34]. The literature reports that depression in clinical populations has been diagnosed in 5 to 10% of outpatients and 9 to 16% of hospitalized individuals [35]. Mood disorders in sickle cell anemia patients have been related to the chronic nature of the disease, unpredictable episodes, physical changes, delay in sexual maturation and restrictions imposed by the treatment [36–38].

### PMQ

The results of this study showed that the participants frequently ask their doctors for a new prescription when their medication runs out early, or borrow drugs from family or friends, corroborating the study of [39]. Pre-prescription refills or increased doses may be much more related to the unpredictability of episodes of chronic and acute pain than to nonconformities [14–17, 40].

Opioid consumption in the study population demonstrated obsessive thinking processes and concentration problems [41, 42], and that the sensation and perception of pain was different in each individual [43]. The results indicated that the study population prefers to use opioids, and nearly half believed they were receiving an insufficient dosage to relieve their pain, similar to the findings of [40]. More frequent consumption of analgesics was associated with greater risk of inappropriate use [44].

It was observed that sickle cell patients, primarily users of opioids and benzodiazepines, often mixed sedatives and analgesics in order to increase the pain-killing effect, and that this combination may have negative consequences, as reported by [45].

9pt?>Some clinical symptoms related to the adverse effects of opioids, such as constipation and nausea, were similar to those found by [46]. Similarly, the unpredictable recurring vaso-occlusive crises experienced by sickle cell patients were associated with pain in various parts of the body, leading to hospitalization and low quality of life, as observed in other studies [18, 47, 48]. Moreover, insomnia in some patients was related to opioid abuse, probably due to the recurrence of chronic pain in this population [49, 50].

The predictors of inappropriate opioid use in this study were similar to those of other investigations, such as disease, substance abuse and pain-related physical limitations [51–53]. Leg ulcers in sickle cell patients increased pain, contributing to low quality of life [54], and are a medium/high risk factor for opioid abuse, according to the PMQ.

The findings demonstrate the complexity of opioid abuse and the importance of measuring it with a valid and reliable instrument. The association with many risk factors, such as sociodemographic characteristics, number of painful lesions, pain intensity, frequency of opioid use, substance intake, personality types and depressive symptoms [55] characterize people with high levels of pain and emotional suffering, as well as complex psychosocial histories [50].

### Internal consistency

Internal reliability was considered adequate, evidenced by Cronbach’s alpha coefficient (0.705), similar to the results of other studies on the PMQ [14–18, 44, 56].

### Strength and limitations

This study adhered to a rigorous cross-cultural adaptation process [19] that provided abundant data and details on the linguistic differences between the original version of the instrument and the one adapted for Brazil, producing scientific evidence confirming that the PMQ can be easily understood by Brazilians with sickle cell anemia.

The authors consider the small sample size (40 participants) a study limitation, despite the fact that it is considered adequate for the cross-cultural adaptation process [57].

## Conclusion

This we intend to exhibit the PMQ’s cross-cultural adaptation results, in a satisfactory level, in terms of language clarity, practical relevance, theoretical relevance, semantic equivalence, idiomatic equivalence, experiential equivalence, conceptual equivalence and good internal consistency. Furthermore, there is a strong correlation between sickle cell anemia and the risk of opioid abuse, with a negative impact on the quality of life of the subjects assessed in Brazil.

The translation and cross-cultural adaptation of the PMQ instrument to Brazilian.

Portuguese is an initial step in implementing an instrument that assesses the risk of opioid abuse in people with heterogeneous chronic pain, fulfilling the need to individualize the treatment of chronic pain in each patient, improve health care quality and the knowledge of health professionals with respect to managing opioid use, plan interventions, monitor the risk of opioid abuse and evaluate the effectiveness of sickle cell anemia treatment in patients using opioids.

This study reinforces the need for constant vigilance of this behavior in patients afflicted by this hemoglobinopathy, and the subsequent use of the instrument by other Brazilian centers will be important to confirm its validity nationwide, considering Brazil’s cultural diversity. It is important to underscore that the same research team will proceed with studies on the issue and that the article on the clinical validation of the PMQ is near completion.

## Additional file

Additional file 1: Pain Medication Questionnaire Scale original version. (DOCX 967 kb)

## Abbreviations

AI: Agreement Index
BT1: One Back translation
BT2: Second Back translation
CAAE: Certificate of Presentation for Ethical Appreciation
HbS: Hemoglobin S
HEMOPE: Blood Center of Pernambuco
IBM: International Business Machines
PMQ: Pain Medication Questionnaire
SPSS: Statistical Package for Social Science
T1: One translator
T12: Synthetized version of the instrument
T2: Second translator
TENS: Transcutaneous Electrical Nerve Stimulation
