# Supplementary material for: Cross-cultural adaptation of the Pain Medication Questionnaire for use in Brazil
Source: BMC Med Res Methodol. 2019 Sep 23;19:188. doi: 10.1186/s12874-019-0821-x (PMC6757373; doi:10.1186/s12874-019-0821-x)
Supplement: Supplementary file 1 — Pain Medication Questionnaire Scale original version. (DOCX 967 kb) [file 12874_2019_821_MOESM1_ESM.docx]

Original Article

Development of a Self-Report Screening

Instrument for Assessing Potential Opioid

Medication Misuse in Chronic Pain Patients

Instrument original Pain Medication Questionnaire


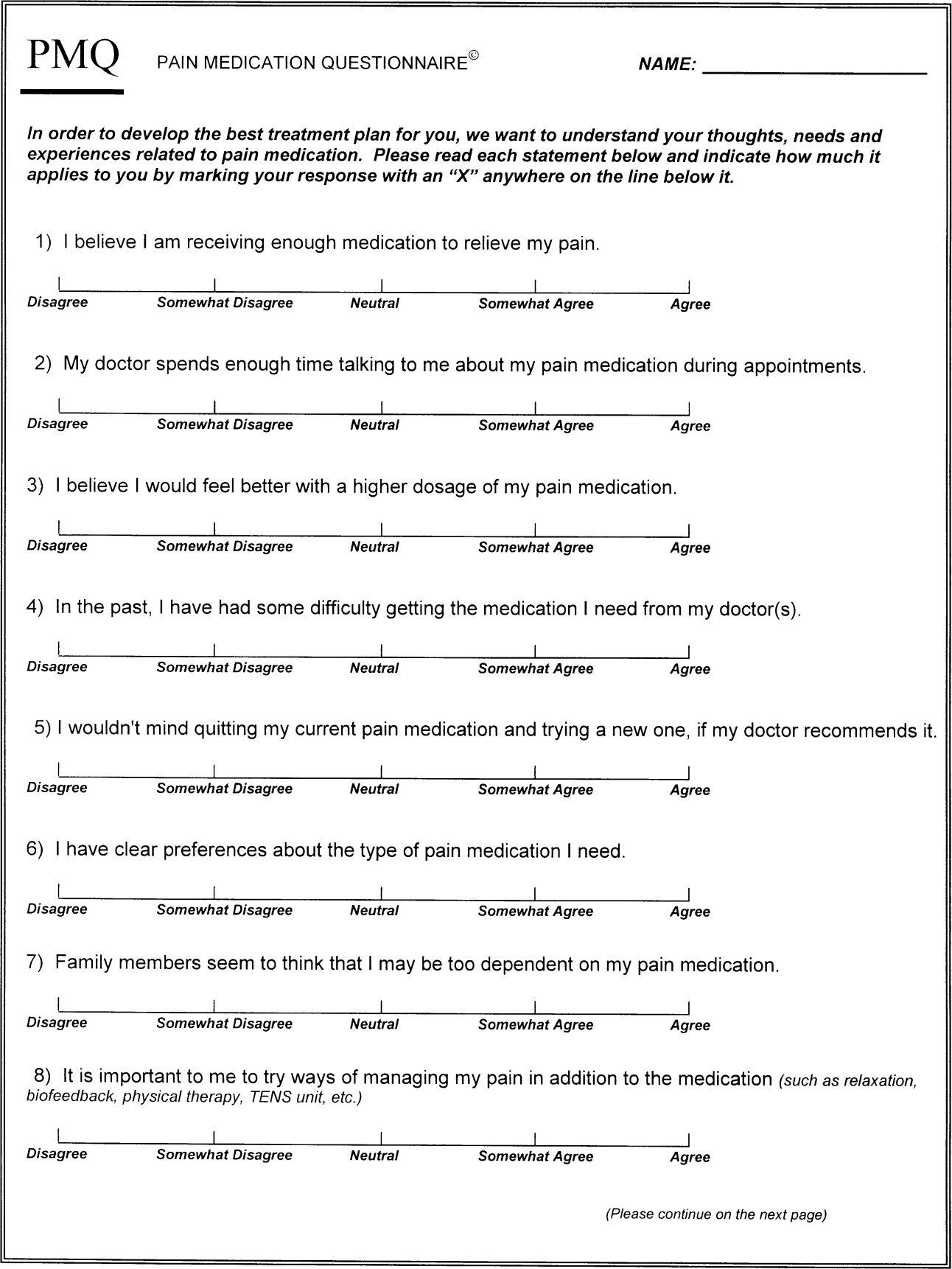


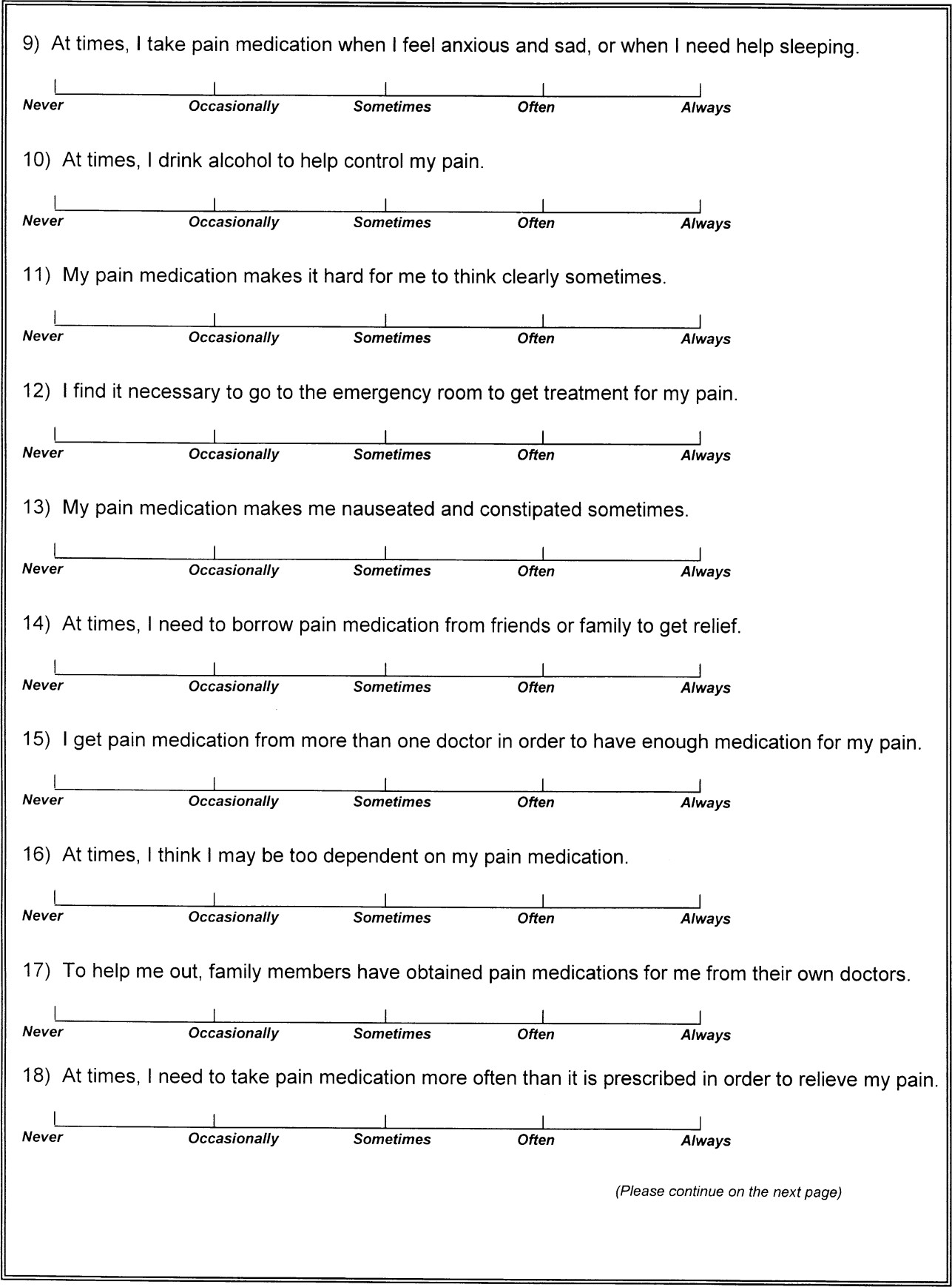


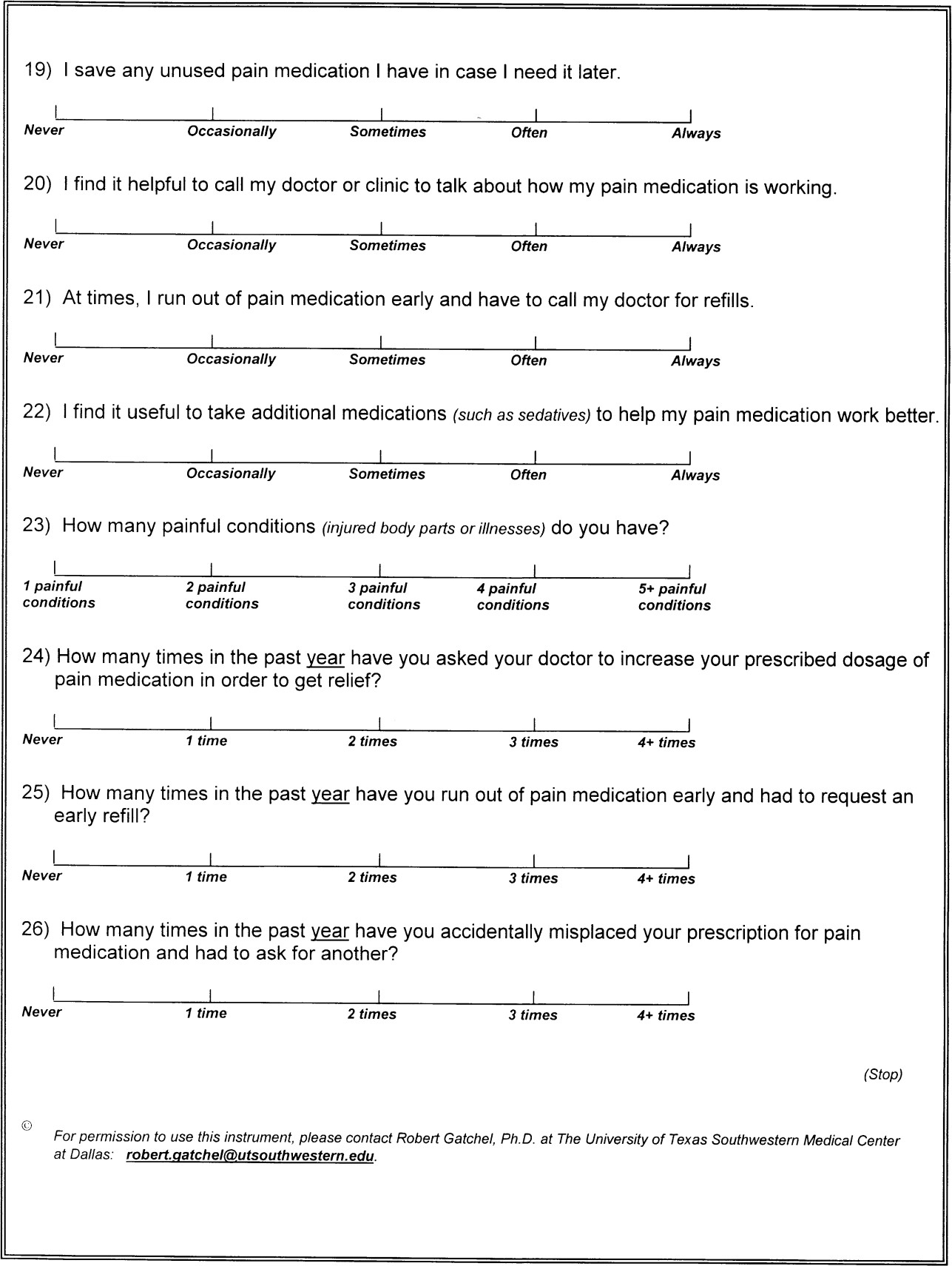


Robert J. Gatchel and Michael Shaffer conducted the revised PMQ-r validation study in 300,000 individuals.

The PMQ can only be used with their permission, which can be obtained at: michael.shaffer@usmedsci.com.

This instrument was authorized by the authors to be validated in Brazil.

ADAMS, L., GATCHEL, R., ROBINSON, R., POLATIN, P., GAJRAJ, N., DESCHNER, M., NOE, C.,2004. Development of a self-report screening instrument for assessing potential opioid medication misuse in chronic pain patients. J. Pain Symptom Manage. 27, 440–459.
